# Exercise in Older Adults to Prevent Depressive Symptoms at the Time of Covid-19: Results of a Randomized Controlled Trial with Follow-Up

**DOI:** 10.2174/17450179-v18-e2112231

**Published:** 2022-03-15

**Authors:** Mauro Giovanni Carta, Cesar Ivan Aviles Gonzalez, Luigi Minerba, Massimiliano Pau, Mario Musu, Fernanda Velluzzi, Caterina Ferreli, Elisa Pintus, Sergio Machado, Ferdinando Romano, Veronica Vacca, Antonio Preti, Giulia Cossu, Laura Atzori

**Affiliations:** 1 Department Medical Sciences and Public Health, University of Cagliari, Cagliari, Italy; 2 Azienda Ospedaliero Universitaria di Cagliari, Cagliari, Italy; 3 Universidad del Cesar, Valledupar, Colombia; 4 Dipartimento di Ingegneria Meccanica, Chimica e dei Materiali, Università degli Studi di Cagliari, Cagliari, Italy; 5 Department of Sports and Methods Techniques, Federal University of Santa Maria (UFSM), Brazil; 6 Laboratory of Physical Activity Neuroscience, Neurodiversity Institute, Queimados- RJ , Brazil; 7 Department of Public Health and Infectious Diseases, Università Roma Sapienza, Roma, Italy; 8 Department of Medical Sciences and Public Health, University of Turin, Turin, Italy

**Keywords:** Physical exercise, Depressive symptoms, Older adults, COVID-19 lockdown, Prevention, Moderate physical activity

## Abstract

**Background:**

This study aimed to verify, through a randomized controlled trial, whether a medium-intensity mixing/aerobic/anaerobic exercise (accessible to older adults even with mild chronic diseases) can effectively counteract depressive episodes. A characteristic of the trial was that the follow-up coincided (unscheduled) with the lockdown due to Covid-19.

**Methods:**

Participants (N=120) were randomized into an intervention group, performing physical exercise, and a control group. Participants, aged 65 years and older, belonged to both genders, living at home, and cleared a medical examination, were evaluated with a screening tool to detect depressive episodes, the PHQ9, at pre-treatment, end of the trial (12-week), and follow-up (48-week).

**Results:**

A decrease in the frequency of depressive episodes after the trial (T1) was found in both groups; however, a statistically significant difference was observed only in the control group (p=0.0039). From T1 to follow-up (conducted during the lockdown), the frequency of depressive episodes increased in the control group, reaching a frequency equal to the time of study entry (p=0.788). In the experimental group, the frequency of depressive episodes did not change at the end of the trial but reached a statistically significant difference compared to the start of the study (p = 0.004) and was higher than the control group (p=0.028).

**Conclusion:**

Moderate-intensity physical exercise can be conducted safely, benefitting older adults even suffering from mild chronic disorders. Physical exercise seems to guarantee a long-term preventive effect towards depressive symptoms, especially in serious stressful situations such as the lockdown due to the Covid-19 pandemic.

**Clinical Trial Registration Number (NCT03858114):**

## 1 INTRODUCTION

Approximately 30% of the European population will be over 65 years of age by 2050. The frequency of chronic diseases increases in late life, and disability-adjusted life years (DALYs) increase proportionally, impacting social and health costs [1]. An active life can counteract the loss of autonomy and disability in older people; the European Union has declared research on active aging as a priority [2, 3].

Physical exercise (PE) lowers the risk of disability and decline in motor function, thus preventing falls, a well-known determinant of early death and disability in older adults [4]. Studies have also shown that PE regulates immune responses in old age, improves metabolic disorders, including diabetes [5], and impacts quality of life [6] as well as cognitive and memory decline [7]. PE can prevent depression [8], in contrast to the use of antidepressants that have certain side effects [4].

International guidelines indicate PE is a preventive factor against cognitive decline and functional limitations in old age [9-11]. However, a recent meta-analysis found no clear evidence on which characteristics of the exercise may be useful (*i.e*., for which problems an aerobic or anaerobic exercise is more suitable or a mix of which two types, whose intensity, duration, and frequency are the best). Studies showed an imbalance with generally smaller control groups and a strong inhomogeneity in control procedures (without placebo, with questionable types of placebo) [12]. The critical point is that clinical trials published so far have been based on intense physical activity (PA), thus excluding participants with mild chronic diseases (such as hypertension, osteo-muscular disorders, and diabetes), which are very frequent in older people, who could benefit most from the preventive effects of physical exercise. Furthermore, most of the studies have adopted programs with the frequency of sessions being 4-5 per week, which is difficult to practice in real life, especially for long periods [12].

It is possible that medium-intensity activity with mixed aerobic/anaerobic exercises may effectively reduce or alleviate depressive symptoms. Thus, a prototype of PA training suitable for older people, as per the weekly frequency of sessions and middle intensity of effort required, with evidence of being safe and effective, could have the greatest impact on mood disorders in older adults. This kind of PA intervention could also be accessible for people with minor chronic diseases common in older adults living in the community (diabetes, hypertension, and so on). Considering this population is at the highest risk of loss of autonomy, and depressive comorbidity plays a fundamental role in this decline [4], an effective preventive measure for depression could drastically impact the well-being of many people, in contrast to the loss of autonomy and institutionalization in facilities for people with disabilities.

This study aimed to verify, through a randomized controlled trial with parallel and balanced groups, whether a medium-intensity physical activity involving aerobic and anaerobic exercise (could be enjoyed by older adults living in the community, even with mild chronic diseases) can effectively counteract depressive episodes and depressive symptoms. An additional characteristic of this trial was that, in an unscheduled manner, the 48-week follow-up coincided with the lockdown due to Covid-19 (April–March 2020). Consequently, there was a certain loss of people who could complete the assessment. On the other hand, it allowed for assessing their mental health during a particularly, stress time, considering how much older people were at risk of more serious consequences from SARS-CoV-2 infection.

## 2 MATERIALS AND METHODS

### 2.1 Design

**Fig. (1):**
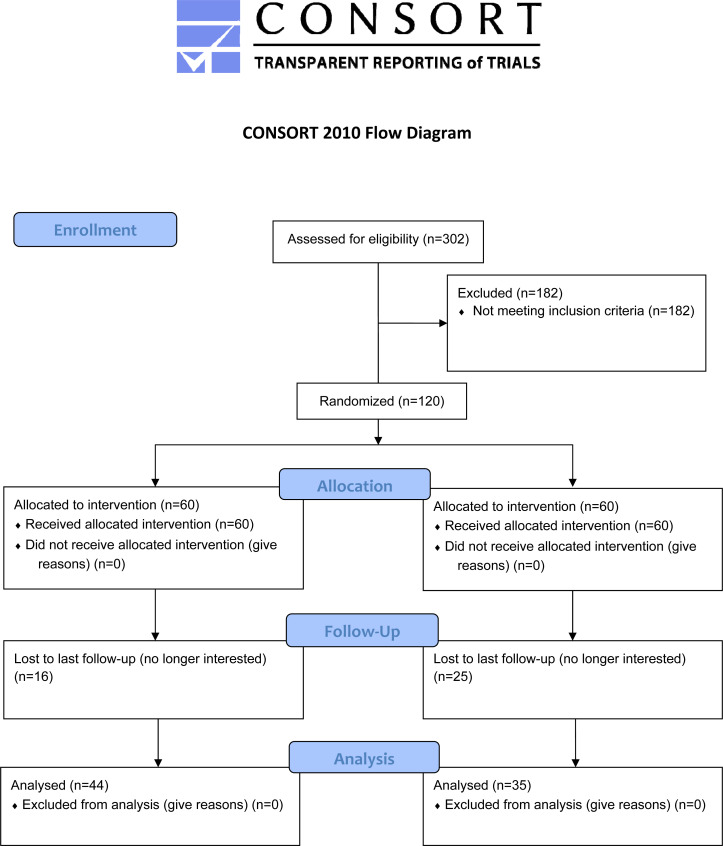
CONSORT flow diagram.

The present study involved a sub-analysis of the data from a 12-week randomized controlled trial (RCT) with follow-up at 48-week (Fig. **1**), whose methods have been described and published in a prior study [13].

Participants (N = 120) were randomized to either an experimental intervention of physical exercise (n = 60) or a control intervention (n = 60) that included group activities focusing on the history of local culture, a visit to the historical sites of the city, and wellness education. Time commitment and the level of social interaction were similar to those in the physical exercise intervention group. Prior to participation, they received information about the study, and informed consent was obtained. All participants were assessed in the time frame of two weeks preceding the intervention (pre-treatment period), at the end of the trial (12-week), and follow-up (48-week). Participants underwent assessments for physical, medical, and psychological conditions during the treatment period. Randomization was carried out using a computer program with blocks at a 1:1 rate; codes were masked after the pre-assessment of the participants. After inclusion in the study, health-trained staff supervised the PA and control interventions. Research evaluators were blinded to the kind of intervention received by each participant.

### 2.2 Study Tool

The Patient Health Questionnaire-9 (PHQ-9) [14] is a self-administered screening tool with high accuracy for detecting depressive episodes. The overall score is the sum of the responses to nine items (coded from 0 to 4), each corresponding to one core symptom of the DSM criteria. An overall score of 5 or more indicates a high probability of an episode of depression (5–9 mild, 10–14 moderate, 15–19 moderately severe, and 20–27 severe) [14].

### 2.3 Participants and Recruitment

Participants were aged 65 years and older, belonged to both genders, and were living at home. People were recruited through local media with the support of the Italian Olympic Committee (CONI) or by the network of general practitioners.

To be eligible for the trial, each participant was required to pass a specialist medical examination at the University of Cagliari to provide a medical certificate for suitability in non-competitive physical activity. At the time of the visit, any present or past illness was recorded.

The exclusion criteria were as follows: a body mass index (BMI) of more than 35, unsuitable for moderate physical activity for health conditions, and involvement in a physical exercise program within the last two years.

### 2.4 Interventions

#### 2.4.1 Physical exercise

The PA intervention was organized into three sessions per week. PA was fixed at 60%–80% of the heart rate reserve (HRR), and it was monitored during the activity and transmitted to professionals using a wireless telemetry apparatus. The individual HRR for each participant was calculated using the estimated maximal heart rate formula. Baseline HR was already registered in the pre-treatment period for each participant as a 3-day mean. PA was carried out in three phases: (a) *warm-up,*10-min, 60% of HRR; (b) *active phase*, 45-min, 60%–80% HRR; (c) *cool down*, 10-min, <60% HRR. The active phase was established as a mixture of aerobic and anaerobic exercises, including drills of “life movements” and balance exercises.

#### 2.4.2 Educational and cultural activities

People in the control group participated in cultural and recreational activities focusing on education, wellness, and the history of local culture. An “animator” (with a degree of educator) carried out the program in groups of the same size (*n* = 15) and time spent, similar to that of the intervention group. The control group condition was conceived to provide a comparator stimulus, while all the confounding effects with the potential impact of depression other than exercise were considered (*e.g*., sociality, amusement, bonding, sharing of time, companionship).

### 2.5 Statistical Analysis

All statistical tests were two-tailed with an alpha set at p < 0.05. Categorical analyses were carried out with the chi-square test, along with Yates correction, or by Fisher’s exact test whenever necessary (*i.e*., presence/absence of depressive episode and PHQ-9 ≥ 5). Means and standard deviations were calculated for continuous variables. If necessary, continuous variables were tested using the Student's t-test or ANOVA for repeated measures.

The comparison by group (experimental *vs*. control) and time (T0 *vs*. T1 in all those who completed at least observation at T1 and T0 *vs*. T1, T0 *vs*. follow-up, and T1 *vs*. follow-up, in all those who remained in the study till follow-up) was made concerning the frequency of the depressive episode (PHQ-9 ≥ 5). Further analysis of the mean frequency of depressive scores was also performed to compare T0 and T1.

## 3 RESULTS

The demographic characteristics and health status at the start of the study of those who completed all observations of the two groups are shown in Table **1**. Forty-four participants (73.3%) in the PE group and 35 participants (58.3%) in the control group were evaluated at follow-up. There were no significant differences between the two groups with regard to age, gender, and the main pathologies. Regardless of age, a considerable portion of the sample had non-negligible chronic pathologies, with only approximately 40% affected with hypertension and about 10% with diabetes.

As shown in Table **2**, in both groups, there was a decrease in the frequency of depressive episodes after the trial; however, differences over time were observed between the two groups: There was a statistically significant difference only in the control group for T0 to T1 (28.6% *vs*. 2.6%, χ^2^ = 8.737, p = 0.003, OR = 13.6, 95% CI [1.6-113.2]). However, the trend in the two groups changed from T1 to follow-up (conducted during the lockdown). In the control group, the frequency of depressive episodes had risen, reaching a frequency of depressive episodes equal at the start of the study (T0 28.6% *vs* 25.7% at follow-up, χ^2^ = 0.072, p = 0.788, OR = 1.1, 95% CI [0.4-3.3]), and a higher frequency compared to the end of the RCT (25.7% at follow-up *vs* 2.6% at the end of the RCT, χ^2^ = 7467, p = 0.017, OR = 0.1, 95% CI [0.1-0.7]). In the PE group, the frequency of depressive episodes did not change at the end of the trial and reached a statistically significant difference compared to the start of the study (18.1% at T0 *vs*. 4.5% at follow-up, χ^2^ = 4.062, p = 0.004, OR = 4.6, 95% CI [1.0-23.9]). At follow-up, the frequency of depressive episodes was lower in the PE group (4.5% *vs*. 25.7%) than the control group (Fisher’s exact test p = 0.028, OR = 0.21, 95% CI [0.05-0.84]).

Table **3** shows the characteristics by age and gender of those who finished the RCT (T1 after 12 weeks) in the PA (exercise) and control (cultural activity) groups. Although slightly more women than men completed the evaluation at T1 than those who participated in the follow-up, no differences were found to be statistically significant. In this evaluation (Table **4**), the differences between T0 and T1 in the frequency of depressive episodes were statistically significant in both the PE group (19.2% at T0 *vs* 5.8% at T1, χ^2^ = 4.308, p = 0.038, OR = 3.88, 95% CI [1.1-15.6]) and the control group (28.6% *vs*. 2.6%, χ^2^ = 10.609, p < 0.001, OR = 15.2, 95% CI [1.9-13.4]), while the comparisons between groups at both T0 and T1 did not show statistically significant differences.

## 4 DISCUSSION

Our study showed that both a cultural activity with an emphasis on sociality and bonding with others and a medium intensity physical exercise (mixed aerobic-anaerobic), similarly carried out for 12 weeks, can decrease the frequency of depressive episodes in a sample of older adults.

Participants in the cultural activity group showed a significant difference at the end of the 12-week trial, even the smallest sample of participants who finished the entire follow-up, while physical activity did not. However, because there was no difference in comparisons between groups at T0 and T1, it could not be concluded that one treatment was superior to the other. Therefore, it can be assumed that the absence of statistical significance in the T0 *vs*. T1 difference in the frequency of depressive episodes in the exercise group is likely due to the low power of the study because of the smaller sample.

A maximum drop-out rate of an RCT of 20% was estimated [13], which was fully considered since the loss was only 12.5%. However, at the follow-up, the percentage of those who could not attend the assessment was very high (28.6%), probably because the 48 weeks since the beginning of the RCT included April till May 2020, during the lockdown due to the Covid-19 pandemic in Italy. This high drop-out rate lowered the power of the study; however, it allowed us to study an unusual stressful framework for older adults [15] and older adults with concomitant health conditions [16, 17]. Thus, the circumstances provided results of unusual interest. It is, in fact, worth noting that although the cultural activity was associated with a decrease in the frequency of depressive episodes to a surprising extent at the end of the trial, at follow-up, during the peak of lockdown, the sample returned to the frequency of episodes equal to the starting point of the study. On the contrary, physical exercise was associated with a very low frequency of depression, even lower than in the initial evaluation and lower than in the control sample in the same evaluation period.

Although taken with great caution given the small size of the sample, the results were not easy to interpret.

First, physical exercise, although completed 36 weeks before the time of the last evaluation, may have caused a better sense of well-being and efficiency. In a situation like the pandemic (and the related lockdown), where people know that in the event of an infection, better physical efficiency can cause an auspicious outcome, it is possible that having conducted a period of physical training may have contributed to a sense of greater self-confidence [18, 19]. It should be noted that the encouragement to continue or start physical activity to improve protection in case of infection was a message provided to the older adults by the social services of the municipality, which therefore might have increased the sense of security in those who had done physical exercise. Moreover, those who completed the training period were probably better able to continue the physical activity, as suggested by social services.

However, there is also an element of non-negligible importance that should be considered. In the results published from the previous research study using the same sample as in the present study, it was evident that physical exercise, but not a cultural activity, induced an improvement in social and behavioral factors (sleep rhythm, nutrition, social contacts) [20]. These changes were maintained for the 48 weeks of assessment [20].

Social and behavioral rhythms are closely related to the circadian rhythms dictated by the light/dark cycle, with melatonin and cortisol as the main pacemakers in the body [21-23]. Not only is it known that social rhythms are a key element in the pathophysiology of mood disorders, but it has also been shown that lockdown can induce changes in social rhythms and expose individuals to the risk of depressive episodes [24]. In the same sample analyzed in the present research, it was also shown that good efficiency of social rhythms was closely associated with resilience concerning depression during lockdown [25].

The data presented in this study seem to indicate that physical exercise carried out months earlier can induce resilience with respect to depression that has lasting effects in older adults. However, given that physical exercise induces an improvement in social rhythms associated with resilience towards depression, it may be assumed that exercise could be mediated by changes in social rhythms.

The present study has certain limitations, such as the sample was not of a big size to verify the expected interactions between physical exercise, modification of social rhythms, and depression. However, the lockdown coincided with the follow-up evaluation of our sample, which likely affected attrition and our results. Nonetheless, the analysis uncovered some very interesting findings considering the Covid-19 pandemic and related lockdown.

## CONCLUSION

Moderate-intensity physical exercise can be conducted safely and benefits older adults suffering from mild chronic disorders such as those frequently occurring in the community. Although benefits with regard to reducing depressive symptoms by engaging in physical activity can also be achieved by socializing activities, only physical exercise seems to guarantee a long-term preventive effect, especially in a serious, stressful situation such as the lockdown due to the Covid-19 pandemic.

## AUTHORS’ CONTRIBUTIONS

The study was initially designed by MGC and GC and then discussed with MP, FV, LA, LM, RD, SM, DF, FM, and CIAG. The methodology was decided by MGC, GC, LA, FR, and GC. The MGC conducted the data analysis. The results were collectively discussed. MGC and GC drafted the paper, and FV and LA revised the manuscript. All authors read and approved the final manuscript.

## Figures and Tables

**Table 1:** Active (Exercise) and Control (Cultural activity) Samples: characteristics and health status at the start of the study; data are expressed as counts (%) or mean (standard deviation).

**-**	**Exercise** **N=44**	**Control Group** **N=35**	**Likelihood and Statistics**
Gender (Women)	23 (42.3%)	19 (47.9%)	χ2=0.04, p=0.838
Age	72.6 (4.6)	72.2 (4.7)	F(1;103)=0.76, p=0.385
Education Years	14.0 (4.3)	13.1 (4.9)	F(1;103)=2.27, p=0.124
Thyroid’s Diseases	10 (27.7%)	4 (11.4%)	χ2=1.86, p=0.179
Chronic respiratory or heart diseases	2 (4.5%)	2 (5.7%)	χ2=0.001, p=0.999 (with yates correction)
Painful sequelae for cancer or fibromyalgia	2 /4.5%)	3 (8.6%)	χ2=0.001, p=0.999 (with yates correction)
Osteoporosis or osteoarthritis	5 (11.4%)	8 (22.8%)	χ2=1.130, p=0.288 (with yates correction)
Hypertension	16 (36.4%)	18 (51.4%)	χ2=3.449, p=0.063
Diabetes 2	3 (6.8%)	5 (14.2%)	χ2=0.581, p=0.466 (with yates correction)

**Table 2:** Comparison of the frequency of depression (PHQ> 4) at T0, T1, and follow-up in the two samples.

**-**	**T0**	**T1**	**Follow-up**	**T0 *vs*.T1**	**T0 *vs*. F-up**	**T1*vs*. F-up**
Exercise (N=44)	8 (18.1%)	3 (6.8%)	2 (4.5%)	χ2=2.597 p=0.107 OR=3.0 CI95% (0.7-12.3)	χ2=4.062 p=0.004 OR=4.6 CI95% (1-23.9)	χ2=0.212 p=0.645 OR=1.53 CI95% (0.2-9.8)
Control group (N=35)	10 (28.6%)	1 (2.6%)	9 (25.7%)	χ2=8.737 p=0.003 OR=13.6 CI95% (1.6-113.2)	χ2=0.072 p=0.788 OR=1.1 CI95% (0.4-3.3)	χ2=7467 p=0.017 OR=0.1 CI95% (0.1-0.7)
-	χ2=1.851 P=0.174 OR=0.47 (CI95%0.16-1.40)	Fisher P=0.625 OR=2.48 (CI95%0.24-25.0)	Fisher P=0.028 OR=0.21 (CI95%0.05-0.84)	-	-	-

**Table 3:** Active (Exercise) and Control (Cultural activity) Samples: characteristics of those who completed the RCT (T1 after 12 weeks).

**-**	**Exercise**	**Control group**	**Statistics**
	**N = 52**	**N = 53**	
Gender Men Women	23 (44%) 29 (56%)	19 (36%) 34 (64%)	χ2=0.77, p=0.381
Age	71.8 (4.7)	72.7 (4.7)	F(1;103)=0.76, p=0.385
Years of education	14.1 (4.6)	12.7 (4.9)	F(1;103)=2.27, p=0.124

**Table 4:** Comparison of the frequency of depressive episode (PHQ score>4) at T0 and T1 in the two samples involving people who completed the first two assessments at the end of CRT.

**-**	**T0**	**T1**	**T0 *vs* T1**
Exercise (N=52)	10 (19.2%)	3 (5.8%)	χ2=4.308 p=0.038 OR=3.88CI95% (1.1-15.6)
Control group (N=53)	12 (22.6%)	1 (1.9%)	χ2=10.609 P<0.001 OR=15.2CI95% (1.9-13.4)
-	χ2=0.184 p=0.668 OR=0.81 CI95% (0.3-2.1)	Fisher P=0.943 OR=3.1 (CI95%0.3-31.6)	-

## Data Availability

The datasets are available only after requests for access directed to project leader Mauro Giovanni Carta as guarantor, according to the agreement shared with the participants and partners, and as stated in the presentation for authorization to the ethics committee.
